# Additive Bayesian networks for antimicrobial resistance and potential risk factors in non-typhoidal *Salmonella* isolates from layer hens in Uganda

**DOI:** 10.1186/s12917-019-1965-y

**Published:** 2019-06-24

**Authors:** Sonja Hartnack, Terence Odoch, Gilles Kratzer, Reinhard Furrer, Yngvild Wasteson, Trine M. L’Abée-Lund, Eystein Skjerve

**Affiliations:** 1 Section of Epidemiology, Vetsuisse Faculty, University of Zurich, Winterthurerstrasse 270, 8057 Zurich, Switzerland; 2 Department of Biosecurity, Ecosystems and Veterinary Public Health, College of Veterinary Medicine, Animal Resources and Biosecurity (COVAB), Makerere University, P.O. Box 7062, Kampala, Uganda; 3 Department of Mathematics, University of Zurich, Winterthurerstrasse 190, 8057 Zurich, Switzerland; 4 Department of Computational Science, University of Zurich, Winterthurerstrasse 190, 8057 Zurich, Switzerland; 5 Department of Food Safety and Infection Biology, Faculty of Veterinary Medicine, Norwegian University of Life Sciences (NMBU), 0454 Oslo, Norway

**Keywords:** *Salmonella*, Antimicrobial resistance, Multi-drug resistance, Patterns of antimicrobial resistance

## Abstract

**Background:**

Multi-drug resistant bacteria are seen increasingly and there are gaps in our understanding of the complexity of antimicrobial resistance, partially due to a lack of appropriate statistical tools. This hampers efficient treatment, precludes determining appropriate intervention points and renders prevention very difficult.

**Methods:**

We re-analysed data from a previous study using additive Bayesian networks. The data contained information on resistances against seven antimicrobials and seven potential risk factors from 86 non-typhoidal *Salmonella* isolates from laying hens in 46 farms in Uganda.

**Results:**

The final graph contained 22 links between risk factors and antimicrobial resistances. Solely ampicillin resistance was linked to the vaccinating person and disposal of dead birds. Systematic associations between ampicillin and sulfamethoxazole/trimethoprim and chloramphenicol, which was also linked to sulfamethoxazole/trimethoprim were detected. Sulfamethoxazole/trimethoprim was also directly linked to ciprofloxacin and trimethoprim. Trimethoprim was linked to sulfonamide and ciprofloxacin, which was also linked to sulfonamide. Tetracycline was solely linked to ciprofloxacin.

**Conclusions:**

Although the results needs to be interpreted with caution due to a small data set, additive Bayesian network analysis allowed a description of a number of associations between the risk factors and antimicrobial resistances investigated.

## Background

Antimicrobial resistance (AMR) is a serious global public health challenge putting the use of antimicrobials in jeopardy as microbes develop resistance to essential antimicrobials [1, 2]. Emergence and spread of AMR, including multi-drug resistance (MDR) in bacteria, are seen increasingly. Gaps in our understanding of the complexity of AMR hampers efficient treatment, precludes determining appropriate intervention points and renders prevention very difficult. There is a growing evidence that use of antimicrobials in food producing animals contributes to AMR in *Salmonella* [3]. Different mechanisms for antibiotic resistance in *Salmonella* isolates have been described [4]. The presence of multiple resistance determinants within bacterial isolates can be described as patterns of AMR. Due to biological and evolutionary mechanisms, different resistance genes might be linked to each other (e.g. if stored on the same plasmid), thus their dissemination is being co-dependent. Therefore, systematic and distinct patterns of specific combinations of AMR (coded into 0 and 1) rather than solely random patterns of AMR might be observed. In the context of evaluating a potential factor for intervention it is of interest to assess systematic statistical co-dependencies between multiple antimicrobial resistances.

The difficulty of assessing the role of relevant risk factors, and therefore defining efficient intervention points, can be (at least partly) explained by the lack of appropriate statistical tools for analysing such complex data. In classical risk factor studies, the multivariable regression techniques typically utilized have their origins in experimental research. Here, the investigator is able to fix all the factors of scientific interest at pre-defined levels – an option not available in observational studies. Additionally, to benefit from a higher statistical power, the investigator will aim to obtain a balanced design. This entails attempting to have similar numbers of individuals in different groups, i.e. similar numbers of individuals are being exposed and non-exposed to different risk factors. In contrast, in observational studies, data are typically non-balanced, unless specifically considered in the sampling plan to assure that equal numbers of individuals are exposed and unexposed. In observational studies with non-balanced data, frequently the issue of sparse data or data separation is encountered. When cross-tabulating binary variables, the resulting 2 × 2 cross tables might have a zero in at least one of the four cells. In this situation, confidence intervals might go to infinity, and classic measures as odds ratios may not be estimable.

In an observational setting, if standard multivariable regression is used for analysing the data, risk factors are presumably interrelated, thus precluding the separation of single risk factors and differentiating between direct and indirect effects. Furthermore, in the context of AMR, the response variable consists of a number of different resistant phenotypes and/or genes, thus necessitating a multivariate approach in contrast to classical risk factor analysis with one single outcome, i.e. healthy or diseased. Most often, data on AMR with multiple patterns are analysed in a descriptive way. To quantify the association between antimicrobials, resistance and susceptibility indices have been proposed, which could also be adapted for multiple resistances, providing also confidence intervals [5, 6].

Additive Bayesian network (ABN) modelling, an approach originating from machine learning and not yet seen widely applied in veterinary epidemiology, appears to be a promising tool for the analysis of multivariate resistance data [7, 8]. Notable examples of ABN analyses are published by [9–12]. Still to the authors’ knowledge no study has yet used ABN for the joint analysis of risk factors and binary (resistant/susceptible) antimicrobial resistance data. ABN results are presented in the form of networks, consisting of nodes, representing the variables, and links, designating the conditional probabilities between the variables of interest. ABN modelling is specifically designed to deal with highly correlated and complex data. It is suitable to disentangle direct from indirect statistical associations and can be understood as a generalisation of generalised linear regression models (GLMs). Thus, in contrast to classical regression approaches, the outcome and the predictors are not defined as such beforehand, but within the network different GLMs applicable to the data at hand are evaluated. ABN modelling is a pure data-driven technique, contrasting other approaches where the model is theory driven such as Structural Equation Modeling [13, 14]. Consequently, the first step in an ABN analysis is to find the optimal or most complex network still supported by the data, based on a metric which is controlling for complexity, allowing for the maximum number of links or associations between all variables included. In a second step, measures are undertaken to adjust for potential overfitting and to trim off links that are not supported by the data, given a specific cut-off.

In applied research with binomial (two states random variables) variables, data separation is a surprisingly common issue. It arises when one predictor predicts perfectly the outcome variable. Similarly, the term sparse data is used when only few observations of a possible combination are present in the dataset. Classical approaches, i.e. logistic regression modelling, often fail to accurately estimate the regression coefficient in this situation. The ABN approach requires to perform regressions between all the possible combinations of the variables. Hence, sparsity of the dataset is a major concern and should be addressed properly [15]. The general approach is to control the likelihood in order to prevent it to become infinite. In a Bayesian framework this could be done using an appropriate prior. Equivalently, it can be done using a bias reduction approach [16].

The aim of this study was to determine if specific risk factors are associated with single AMRs and if specific AMRs are linked to each other. For this study we used a data set from a previous study [17].

## Methods

### Sample collection and identification

Non-typhoidal *Salmonella* isolates used in this study were isolated from poultry fecal samples from three districts in Uganda. All flocks were sampled once. The study design and sampling is described in full and reported in [18]. In total 86 isolates originated from 43 farms. Furthermore, the samples were distributed rather homogeneously with 16 farms providing one resistant isolate, 14 farms with two resistant isolates, 10 farms with three resistant isolates and 3 farms with four resistant isolates. A standardized sampling scheme was adapted from previous studies. Culture and isolation followed ISO 6579:2002/Amd 1:2007 Annex D: Detection of *Salmonella* spp. in animal faeces and in environmental samples from the primary production [19]. These analyses were carried out at the food microbiology laboratory at the College of Veterinary Medicine, Animal Resources and Biosecurity, Makerere University, Kampala Uganda. The isolates were serotyped at the Norwegian Veterinary Institute, Oslo, using Kauffman**–**White**–**Le**–**Minor technique [20].

### Antimicrobial resistance testing

Phenotypic antimicrobial susceptibility testing was performed using the Kirby-Bauer disk diffusion methods on Muller-Hinton agar and is described in detail in [18]. The antibiotics were selected based on those commonly used in Uganda and those recommended by World Health Organization (WHO) for routine monitoring and surveillance.

### Statistical analysis: additive Bayesian networks

The following seven risk factors were selected to be included in the ABN analysis: 1) Gender of the manager (binomial, baseline male or female), 2) “Pets”, presence of pets (binomial, baseline no or yes), 3) “Farm size” of the poultry farm (multinomial, baseline small with less than 500 birds, medium between 500 and 1000 birds and large with more than 1000 birds), 4) “Management”, i.e. management practice (binomial, baseline free-range to semi-intensive or intensive), 5) “Eggtrays”, indicating if the egg trays were re-used (binomial, baseline no or yes), 6) “Vaccinator” describing who vaccinates (multinomial, baseline “private service”, “self or family member” or “employee”), 7) “Disposal” of dead birds (multinomial, baseline “burrying”, “burning”, “throw away”, “giving to animals (dogs and pigs)”, and “drop in a pit”). Data on antimicrobial resistance against the following seven different antibiotics ampicillin (AMP), chloramphenicol (CHL), ciprofloxacin (CIPR), sulfamethoxazole/trimethoprim (SXT), sulfonamide (SULFA), tetracycline (TET), and trimethoprim (TRIM), were included as binary variables (baseline no resistance).

The entire statistical analysis was conducted using R [21]. As ABN requires a complete dataset, under the assumption of missing at random, missing values were imputed with the R package *missforest* [22]. ABN analysis was performed with the R package *abn* [23]. Here, a scoring procedure (BIC, Bayesian information criterion) is implemented to identify the maximum a posteriori Bayesian network based on information theoretic metrics [15] and controls internally for model complexity. Estimations of the effect size was done with the function *fitabn.mle()* in the ABN package which is essentially a wrapper for the *multinom()* function in [24] for multinomial random variables. Additionally, for the purpose of comparison and if estimation of standard errors was not stable, the function bayesgln() from the arm package [24] was used. The latter uses as default a student distributed prior that help estimation with sparse dataset [25]. We used an exact search [26] to find first an optimal network, meaning the optimal level of complexity in terms of the simultaneous presence of different GLMs with potential covariates in the data at hand. In this approach, networks of different increasing complexity, i.e. allowing for more links or covariates to be included, were evaluated. For a plausibility check, the magnitude of the marginal likelihood for each model, i.e. individual GLMs, in the network was assessed visually. In order to adjust for overfitting, a non-parametric bootstrapping analysis with 10′000 bootstraps was performed. This means that a part of the data (95% thereof) was randomly selected, then the entire procedure to find the best network was applied. With the aim to obtain robust results, i.e. associations or links between variables being highly supported by the data, a 50% threshold was applied.

## Results

### Descriptive analysis of risk factors and pattern of antimicrobial resistance

In Table 1, the proportions of the seven included risk factors are presented together with the frequencies of susceptible and resistant isolates per antibiotic tested. Antimicrobial resistance testing of 86 isolates originating from 43 farms resulted in 11 different patterns of antimicrobial resistance (Table 2). When looking at the resistance patterns which are at least present with a frequency of *n* = 10, at least 76% originate from different farms. This renders a large clustering effect at farm level implausible in this data set, possibly due to sampling. Out of the 14 farms with two isolates, in seven farms one single pattern was detected and in the other 7 farms there were two distinct patterns. Among the 10 farms with three resistant isolates, in one farm all isolates shared the same single pattern, in seven cases there were two patterns and in 2 farms there were three different patterns. For the 3 farms providing 4 isolates, 2 farms had two patterns and 1 farm had 3 distinct patterns. While 32 isolates (37.2%) were not resistant to any of the seven antibiotics tested, 27 isolates (31.4%) showed resistance against one antibiotic, 16 isolates (18.6%) against two antibiotics, 9 isolates (10.5%) against three antibiotics and 2 against four antibiotics (2.3%). In descending order, the following percentages of isolates were found to be resistant against antibiotics (95% binomial confidence intervals based on Jeffreys approximate method) [27]: ciprofloxacin 46.5% (36 to 58), sulfonamide 24.4% (16 to 34), tetracycline 15.1% (0 to 30), trimethoprim and trimethoprim-sulfamethoxazole both 7.0% (0 to 20), chloramphenicol and ampicillin both 4.6% (1 to 10).

**Table 1:** Descriptive analysis of risk factors analysed classified by antimicrobial resistance

		AMP^a^		CHL^b^		CIPR^c^		SULFA^d^		SXT^e^		TET^f^		TRIM^g^			
Risk factor		S^h^	R^i^	S	R	S	R	S	R	S	R	S	R	S	R		Proportion
(NA^k^)																Total per risk factor	95% CI^j^
	Total	82	4	82	4	46	40	65	21	80	6	73	13	80	6	n	
Proportion of resistance	95% CI^1^	0.05 [0.01;0.1]		0.05 [0.01;0.1]		0.46 [0.36;0.58]		0.24 [0.16;0.34]		0.07 [0.0;0.2]		0.15 [0;0.3]		0.07 [0.0;0.2]			
Gender	Male	58	1	58	1	30	29	41	18	54	5	52	7	54	5	59	0.69 [0.58; 0.78]
(0)	Female	24	3	24	3	16	11	24	3	26	1	21	6	26	1	27	0.31 [0.22; 0.42]
Pets	Yes	28	1	28	1	13	16	20	9	27	2	23	6	27	2	29	0.34 [0.24; 0.44]
(0)	No	54	3	54	3	33	24	45	12	53	4	50	7	53	4	57	0.66 [0.56; 0.76]
Farm size^l^	Small	29	2	29	2	19	12	27	4	30	1	29	2	30	1	31	0.36 [0.26; 0.47]
(2)	Medium	18	0	18	0	10	8	15	3	18	0	17	1	18	0	18	0.21 [0.13; 0.31]
	Large	35	2	35	2	17	20	23	14	32	5	27	10	32	5	37	0.43 [0.32; 0.64]
Management	Free/ Semi	25	2	25	2	16	11	22	5	26	1	25	2	26	1	27	0.31 [0.22; 0.42]
(1)	Intensive	57	2	57	2	30	29	43	16	54	5	48	11	54	5	59	0.69 [0.58; 0.78]
Reuse Egg trays	Yes	31	2	31	2	15	18	25	8	30	3	30	3	30	3	33	0.38 [0.28; 0.49]
(3)	No	51	2	51	2	31	22	40	13	50	3	43	10	50	3	53	0.62 [0.51; 0.72]
Vaccinator	Private Service	30	0	30	0	20	10	26	4	30	0	22	8	30	0	30	0.35 [0.25; 0.46]
(0)	Self or family	35	3	35	3	21	17	31	7	37	1	34	4	37	1	38	0.44 [0.34; 0.55]
	Employee	17	1	17	1	5	13	8	10	13	5	17	1	13	5	18	0.21 [0.13; 0.31]
Disposal	Burrying	45	1	45	1	23	23	31	15	42	4	37	9	42	4	46	0.53 [0.42; 0.64]
(0)	Burning	11	2	11	2	7	6	12	1	13	0	13	0	13	0	13	0.15 [0.08; 0.24]
	Throwing away	19	1	19	1	11	9	15	5	18	2	16	4	18	2	20	0.23 [0.15; 0.33]
	Giving to animals	4	0	4	0	4	0	4	0	4	0	4	0	4	0	4	0.05 [0.01; 0.1]
	Drop in a pit	3	0	3	0	1	2	3	0	3	0	3	0	3	0	3	0.03 [0.00; 0.1]

^a^ Ampicillin

^b^ Chloramphenicol

^c^ Ciprofloxacin

^d^ Sulfonamide

^e^ Sulfamethoxazole/Trimethoprim

^f^ Tetracycline

^g^ Trimethoprim

^h^ Susceptible

^i^ Resistant

^j^ Binomial confidence intervals based on Jeffreys approximate method with a beta distribution

^k^ Missing values which were imputed with the R package *missForest*

^l^ Small 50–500 birds, medium 501–1000, large > 1000 birds

**Table 2:** Descriptive analysis of patterns of antibiotic resistance

Pattern	Antibiotic resistances (0 = susceptible, 1 = resistant*)							Frequencies of isolates per resistance pattern	Frequencies of resistances per pattern	Number of farms per resistance pattern
ID	SULFA^1^	CIPR^2^	TET^3^	TRIM^4^	SXT^5^	CHL^6^	AMP^7^	n	n	n
1	0	0	0	0	0	0	0	32	0	30
2	0	1	0	0	0	0	0	17	1	13
3	1	1	0	0	0	0	0	12	2	10
4	0	0	1	0	0	0	0	8	1	7
5	0	1	0	0	0	1	1	4	3	4
6	0	1	1	0	0	0	0	3	2	2
7	1	0	0	1	1	0	0	3	3	3
8	1	0	0	0	0	0	0	2	1	2
9	1	1	0	1	1	0	0	2	4	2
10	1	1	1	0	0	0	0	2	3	2
11	0	0	0	1	1	0	0	1	2	1

*According to CLSI

^1^ Sulfonamide, ^2^ Ciprofloxacin, ^3^ Tetracycline, ^4^ Trimethoprim, ^5^ Sulfamethoxazole/Trimethoprim, ^6^ Chloramphenicol, ^7^ Ampicillin

In this table summary statistics of the eleven distinct patterns of antimicrobial resistances, based on specific combinations of being susceptible for or resistant against one of the seven antimicrobials investigated are presented. The number of antimicrobial resistances per isolate range from one to a maximum of four. Thus, 32 isolates showed no resistance to any of the seven antibiotics tested, 27 to at least one antibiotic, 16 to two antibiotics, 9 against three antibiotics and 2 against four antibiotics. Additionally the number of farms from which isolates with specific resistance patterns were samples are presented. The four isolates which were resistant against ampicillin originate from four different farms

### Additive Bayesian networks

The results of the final adjusted network are presented graphically, in a table indicating the direction of the associations found (Table 3), as well as numerically with odds ratios on the log.odds and odds scale and standard errors for binomial and multinomial variables (Table 4). In the case of the latter ones, assuming three levels (e.g. vaccination performed by a private service, oneself or a family member, employee) the resulting estimated are referring to the corresponding baseline values.

**Table 3:**
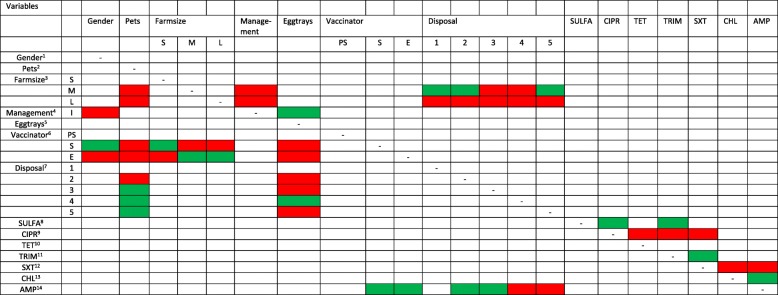
Results of additive Bayesian network. The colors represent the direction of the association with green indicating a positive and red a negative association. The parents are listed in the columns and the children in the rows

^1^Gender (baseline male versus female); ^2^ Presence of pets (baseline no versus yes); ^3^ Farmsize (baseline S: small < 500, M: medium 500 to 1000 and L: large > 1000), M and L compared to S

^4^Management (baseline free range and semi-intensive versus intensive); 5 Eggtrays re-use (baseline no versus yes); ^6^ Vaccinator (baseline PS: private service, S: self or family member, E: employee), S and E compared to PS; ^7^ Disposal (baseline 1 = burrying, 2 = burning, 3 = throwing away, 4 = giving to animals (dogs and pigs), 5 = drop in a pit); ^8^ Sulphonamides, ^9^ Ciprofloxacin, ^10^ Tetracycline, ^11^ Trimethoprim, ^12^ Sulfamethoxazole-trimethoprim, ^13^ Chloramphenicol, ^14^ Ampicillin

The colors in the table are interpreted as follows: female manager compared to male manager are less likely to manage an intensive farm compared to a free-range or semi-intensive farm. A female compared to a male manager is more likely to do the vaccinations by herself or a family member compared to a private service. A female manager compared to a male manager is less likely to have an employeee doing the vaccinations compared to do the vaccination herself or a family member

**Table 4:** Estimated parameters on the log.odds and odds scale for all 22 arcs based on the exact search

child	parent	Effect size	Standard Error	Effect size
		Log.odds	Log.odds	odds
Farmsize M	Pets	−12.6	> > ^u^	3.37E-06
Farmsize M	Management I	−9.6	> > ^u^	6.77E-05
Farmsize M	Disposal 1	34.5	> > ^u^	9.62E+ 14
Farmsize M	Disposal 2	77.1	> > ^u^	3.05E+ 33
Farmsize M	Disposal 3	−23.3	> > ^u^	7.60E-11
Farmsize M	Disposal 4	−65.9	> > ^u^	2.40E-29
Farmsize M	Disposal 5	0.001	1.41	1.00E+ 00
Farmsize M	Pets	−66.9	0.01	8.82E-30
Farmsize L	Management I	−0.4	0.91	6.70E-01
Farmsize L	Disposal 1	−23.6	0.15	5.63E-11
Farmsize L	Disposal 2	−21.9	> > ^u^	3.08E-10
Farmsize L	Disposal 3	− 99.6	1.01E-07	5.55E-44
Farmsize L	Disposal 4	−76	NaN	9.85E-34
Farmsize L	Disposal 5	− 109.1	5.54E-08	4.15E-48
Management I	Gender	−1.9	0.59	1.50E-01
Management I	Eggtrays	2.0	0.58	7.39E+ 00
Vaccinator S	Gender	3.9	1.07	4.94E+ 01
Vaccinator S	Pets	−19.3	> > ^u^	4.15E-09
Vaccinator S	Farmsize S	0.9	0.90	2.46E+ 00
Vaccinator S	Farmsize M	−20.3	> > ^u^	1.53E-09
Vaccinator S	Farmsize L	−2.9	1.08	5.50E-02
Vaccinator S	Eggtrays	−8.7	> > ^u^	1.67E-04
Vaccinator E	Gender	−2.7	1.40	6.72E-02
Vaccinator E	Pets	−17.6	1.42E-07	2.27E-08
Vaccinator E	Farmsize S	−7.4	1.97	6.11E-04
Vaccinator E	Farmsize M	36.8	> > ^u^	9.59E+ 15
Vaccinator E	Farmsize L	3.6	1.29	3.66E+ 01
Vaccinator E	Eggtrays	−26.9	> > ^u^	2.08E-12
Disposal 2	Pets	−0.4	0.80	6.70E-01
Disposal 2	Eggtrays	−2.9	0.74	5.50E-02
Disposal 3	Pets	13.2	> > ^u^	5.40E+ 05
Disposal 3	Eggtrays	−0.2	1.30	8.19E-01
Disposal 4	Pets	1.4	0.74	4.06E+ 00
Disposal 4	Eggtrays	2.5	0.83	1.22E+ 01
Disposal 5	Pets	11.6	> > ^u^	1.09E+ 05
Disposal 5	Eggtrays	−157.8	NaN	2.94E-69
SULFA	CIPR	2.5	0.79	1.22E+ 01
SULFA	TRIM	4.2	1.32	6.67E+ 01
CIPR	TET	−0.4 (−0.4*)	NA	6.70E-01
CIPR	TRIM	−0.3 (− 0.3*)	NA	7.41E-01
CIPR	SXT	−0.3	0.86	7.41E-01
TRIM	SXT	8.9	3.99	7.33E+ 03
SXT	CHL	−3.6 (−0.5*)	NA	2.73E-02
SXT	AMP	−3.6	6.61	2.73E-02
CHL	AMP	8.9	4.83	7.33E+ 03
AMP	Vaccinator S	8.6	1.21	5.43E+ 03
AMP	Vaccinator E	8.7	1.29	6.00E+ 03
AMP	Disposal 2	1.6	1.36	4.95E+ 00
AMP	Disposal 3	0.6	1.23	1.82E+ 00
AMP	Disposal 4	−7.5	2.89	5.53E-04
AMP	Disposal 5	−6.5	3.20	1.50E-03

> > ^u^ indicates that standard errors were so large (i.e. > xE+ 01) that they are not useful. * When the estimation of the standard errors was unstable, presumably due to sparse data, the effect sizes were also estimated with the bayesglm() function for the binomial variable. Table 4 presents the estimated parameters (effect sizes and standard errors) on the log.odds and on the odds scale for all 22 arcs based on the exact search and the function multinom() in [24].

**Fig. 1:**
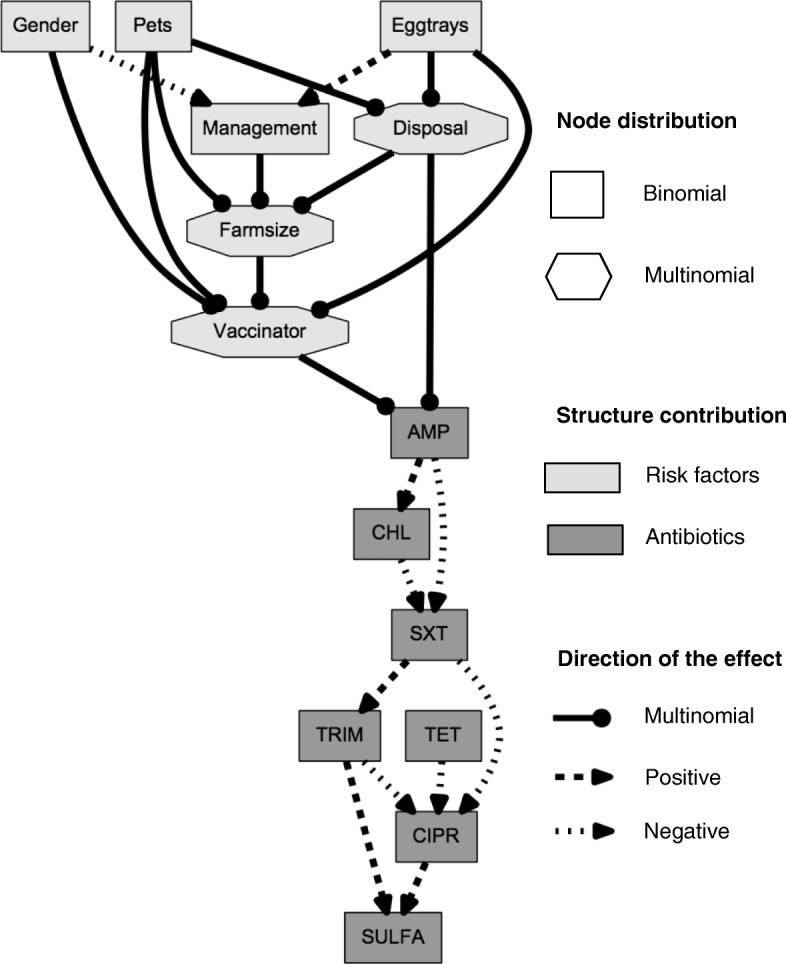
Final Bayesian network graph. Presentation of the variables (antimicrobial resistances and risk factors) with positive or negative associations (dotted lines) between them

**Fig. 2:**
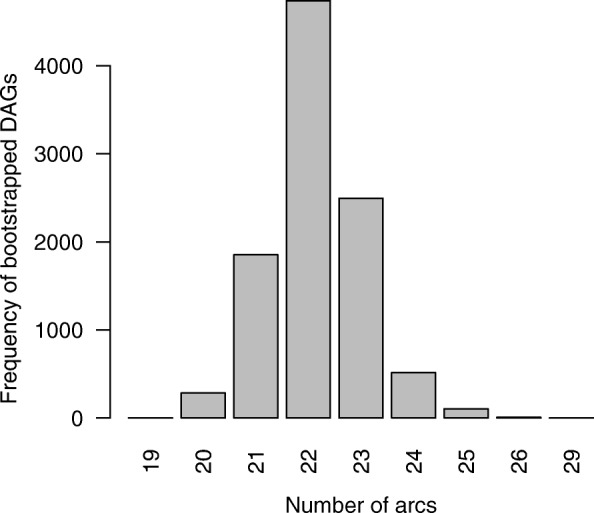
Results of bootstrap analysis: number of arcs in 10,000 bootstrapped networks. The network resulting from the exact search contained 22 arcs. Out of the 10,000 bootstrapped networks more than a third contained more than 22 arcs. This indicates that substantial randomness was introduced and let to overfitting, i.e. spurious arcs and confirms the robustnest of the network with 22 arcs

Six missing values (farm size *n* = 2, management *n* = 1, egg trays = 3) were imputed. The networks before and after bootstrapping are identical with 22 links contained (shown in Fig. 1). Thus, no arcs were pruned. In Fig. 2, the results of the bootstrapping, i.e. the number of arcs in the bootstrapped networks are presented. Based on the number of networks containing more than 22 arcs, corresponding to approximately 31% of the bootstrapped networks, it becomes evident that randomness was actually included by non-parametric bootstrapping and underlines the robustness of the network with 22 arcs.

Regarding the associations between risk factors and antibiotic resistance, solely ampicillin was found to be linked to vaccinator and disposal. Here, ampicillin resistance was more likely, i.e. with a positive log-odds, to occur if vaccination was done by the manager him- or herself and by an employee compared to a private service. Still this needs to be interpreted with caution as there were only four isolates with ampicillin resistance which are of the same pulsotype [17]. These isolates originate from four different farms in two districts.

The following antimicrobial resistance characteristics were linked to each other: resistance towards trimethoprim was linked positively to resistance towards sulfonamide and sulfamethoxazole/trimethoprim, but negatively to ciprofloxacin. Resistance towards sulfonamide was also linked positively to resistance to ciprofloxacin. There was also a positive association between resistance to chloramphenicol and ampicillin, with all isolates being either both susceptible or resistant (*n* = 4). Resistance to ampicillin and to sulfamethoxazole-trimethoprim were negatively associated. There were negative associations between chloramphenicol and sulfamethoxazole/trimethoprim, which was also negatively associated with ciprofloxacin. Tetracycline was also negatively associated with ciprofloxacin.

Regarding the associations between the seven risk factors: intensively managed farms were more likely to have a male compared to a female manager. Female manager compared to male manager were more likely to doing the vaccinations by herself or a family member instead of a public service or by an employee. Medium and large size farms were less likely to have pets compared to small size farms. Intensively managed farms were more likely to reuse egg trays compared to free range or semi-intensive farms.

In Table 4 the corresponding coefficients on a log-odds and an odds scale of the graph before bootstrapping are displayed. Relatively large or small log-odds values and standard errors are indicative of sparse data (at least one zero in a contingency table) with leads to unstable estimation of the effect size. Although the magnitude of the effect size is not necessarily meaningful, the direction of the association is still relevant. For binomial variables, in case the function multinom() did not yield stable standard error estimates, the results of the bayesglm() function are also shown. In all cases, there is agreement about the direction of the association, being positive or negative.

## Discussion

Based on the data from the previously published data [18], despite the presence of sparse data and data separation, it was possible to obtain networks including seven potential risk factors and seven antibiotic resistances. Due to sparse data, the results need to be carefully interpreted. Only resistance to ampicillin was found to be linked directly to the vaccinating person and disposal.

It is a well-known fact that many of the genes coding for AMR characteristics are located on mobile genetic elements, and that these genes are disseminated between related and unrelated bacteria through horizontal gene transmission mechanisms. However, we do not have any data on the location of the genes encoding the AMR characteristics in the bacterial isolates analysed in this study, and can therefore only speculate that one explanation for the AMR linkages observed in the ABN analysis is the physical linkage of genes on the same mobile genetic element. What we do know from the Odoch et al. 2018-study, is that six *S*. Hadar isolates harbored class1 integron genes (*int1*) that were also associated with the gene determinant *dfrA15* encoding trimethoprim resistance. As *int1* always are associated with the *sul1* determinant encoding for sulfonamide resistance, this *int1-sul1-dfrA15* linkage is a molecular explanation for the observed association. Use of antimicrobials is a main driver for development and dissemination of AMR, and the very often standard simultaneous administration of trimethoprim and sulfonamides (trimethoprim-sulfamethoxazole) can probably be regarded as an important driver for evolution of this genetic linkage.

The use of chloramphenicol is banned in poultry, still four isolates were found to be resistant, and the underlying source and mechanisms are unclear. An earlier study identified chloramphenicol resistance encoding gene*, cmlA* in one of these isolates [17]. This requires further investigations.

To our knowledge the only two studies that relied an ABN for analysis on antimicrobial data are Hidano et al. (2015) and Ludwig et al. (2013) [10, 11]. In both studies, not binary data (being resistant or not) but continuous data, assumed to be Gaussian, as zones of inhibition measured in mm were considered. In our study, due to recent adaptions in the abn code, it was possible to directly include the dichotomized antimicrobial resistance data, based on CLSI, without encountering the issue of sparse data. Still due to sparse data, inevitably present in a small data set, not all associations were estimable resulting in very large estimates and standard errors, still with two different approaches, there was agreement about the direction of the association. Another novelty lies in the opportunity to also include multinomial data.

## Conclusions

Although, due to the small sample size and the relative low proportion of resistances against some antimicrobials, the results need to be considered carefully, we are confident, that the actual version of ABN allows for valuable insights in future analyses of larger data sets. The particular added value lies in the opportunity to disentangle the role of single risk factors on the multivariate outcome of antimicrobial resistance data.

## Data Availability

The dataset from which these results were generated are not publically available at this point as this study is part of an on-going PhD research at Norwegian University of Life Sciences and the university takes responsibility of storing the primary data. But this can be made available on reasonable request from the second author.

## Abbreviations

ABN: Additive Bayesian network
AMP: Ampicillin
AMR: Antimicrobial resistance
CHL: Chloramphenicol
CIPR: Ciprofloxacin
GLM: Generalised regression model
MDR: Multi-drug resistance
SULFA: Sulfonamide
SXT: Sulfamethoxazole/trimethoprim
TET: Tetracycline
TRIM: Trimethoprim
